# Robust Control of a New Asymmetric Teleoperation Robot Based on a State Observer

**DOI:** 10.3390/s21186197

**Published:** 2021-09-15

**Authors:** Baoyu Shi, Hongtao Wu, Yongfei Zhu, Mingming Shang

**Affiliations:** 1 College of Mechanical and Electrical Engineering, Nanjing University of Aeronautics and Astronautics, Nanjing 210016, China; sytb@ahut.edu.cn; 2 School of Mechanical Engineering, Anhui University of Technology, Maanshan 243032, China; zyf19950410@163.com (Y.Z.); smm1195747947@163.com (M.S.)

**Keywords:** haptic device, asymmetric teleoperation, baxter, robust control, state observer

## Abstract

This study is mainly about the designation of a new type of haptic device and an asymmetric teleoperation robot system. Aiming at the problems of tracking and transparency of an asymmetric teleoperation system, a robust control algorithm based on a state observer was proposed. The Haptic Device was designed and was chosen as the master-robot of the system. The Baxter dual-arm robot was chosen as the slave-robot of the system. The simulation experiment of robust control based on a state observer of the asymmetric teleoperation robot was carried out. The experiment results showed that the maximum values of displacement tracking errors in three directions x, y, and z are 0.02 m, 0.01 m, and 0.015 m, respectively. Compared with single- joint PID control, the performance of the new control algorithm is improved. The force feedback experiment on the real asymmetric teleoperation robot system was carried out. The results showed that the force feedback wave is consistent with the actual situation and showed that the robust control algorithm proposed is superior to PID. Therefore, the algorithm perfectly satisfied the system. The experiment parameters also demonstrate that the haptic device satisfies the design requirements of the asymmetric teleoperation robots system and the industry standards.

## 1. Introduction

With the development of virtual reality technology, haptic devices play an important role. As an indispensable part of interactive exploration system, haptic devices can achieve the information transmission of motion and force between human and virtual environments; from the perspective of configuration, haptic devices can be divided into series structures and parallel structures. The Hapton Virtuose introduced in the literature [1] is a series structure which is widely used as force feedback equipment and was developed by Hapton, France [1]. It is a 6-DOF series haptic device, and the closed chain structure is adopted in a parallel mechanism, which has higher structural stiffness and a greater load-carrying capacity. However, the disadvantage of joint error accumulation of series mechanisms is abandoned: it is high positioning accuracy [2]. These superior performances make the parallel mechanism meet structural requirements of force feedback equipment; the 6-DOF haptic device introduced in the literature [3] was developed by Niigata university, Japan. It has a compact structure and is used on a desktop platform. A 6-DOF haptic device based on the stewart platform was developed in 2004 in Jilin University [4]. The whole mechanism is driven by hydraulic pressure and equipped with a 6-axis force sensor, which can output large feedback force. However, the isomorphic mapping cannot be achieved and control is simple. Moreover, the haptic device needs a matching hydraulic source, and the overall volume of the equipment is large, so it is not suitable for desktop application. The Harbin institute of technology developed a haptic device for minimally invasive abdominal surgery in 2010 [5]. The haptic device is equipped with a 3-RUU mechanism to achieve position movement, and three rotating pairs are connected in series. It has a compact structure, small inertia, and good reverse drive. However, it can only achieve the force feedback in the moving direction, but not the torque feedback.

**Figure 1:**
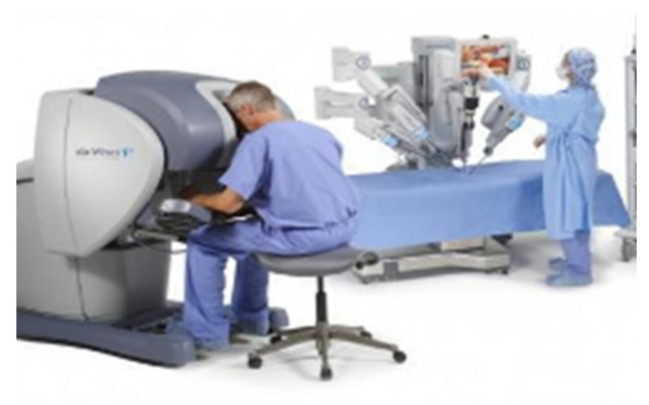
“Da Vinci” surgical robot.

Master–slave teleoperation systems are usually used in various dangerous environments, such as space exploration and nuclear operation. Teleoperation virtual platforms allow people to send their skills and capacities into machines located in either relatively close (a few meters away) or far (different continents) locations [6]. Contact-driven tasks, such as surface conditioning operations (wiping, polishing, sanding, etc.), are difficult to program in advance to be performed autonomously by a robotic system, especially when the objects involved are moving [7]. This work develops an advanced teleoperation and control system for industrial robots in order to assist the human operator to perform the mentioned tasks. They also expand or extend the ability of some special professionals to complete some complex professional and technical works through remote control, such as operations for patients in remote areas. This type of master–slave teleoperation system can be divided into isomorphism and asymmetric. For the Rokviss teleoperation robot, which was developed in Germany, its main application task is to complete the international space station and perform some ground experiments [8,9]. The Da Vinci surgical robot system is shown in Figure 1; doctors can use the “Da Vinci” surgical robot system to perform minimally invasive surgery on patients [10]. Two devices using the 3R and DELTA mechanisms, respectively, are developed to be manipulated to control the position and orientation of a large-sized slave robot by using both of a user’s two hands, respectively [11]. They are isomorphism teleoperation systems. The asymmetric teleoperation system has a wider range of application than that of the isomorphism teleoperation system [12,13,14,15,16].

The performances of master–slave teleoperation systems are focused on stability, tracking, and transparency. In recent years, some new research and methods on master–slave teleoperation systems have been presented. B. Hannaford conducted the research of contradiction between transparency and stability of teleoperation systems [17,18]. Zhang conducted the research that the sliding mod control of teleoperation systems ensures the robustness of the system [19,20]. Khatib performed the research of the time-delay compensation method based on passive theory, and scattering theory is used to ensure the stability of teleoperation robot systems [21]. These studies only considered the stability of isomorphism master–slave teleoperation systems; the tracking and transparency were missed, and the problem of generality was missed.

A delay-dependent control strategy for bilateral teleoperation systems in the presence of passive and constant input forces was proposed by S. Islam et al. [22] in which undelayed position and velocity signals are combined with nonlinear adaptive control terms to deal with the parametric uncertainties associated with the dynamical model of the master and slave manipulator, but the controller system composed of 2-DOF master and slave haptic manipulators was a little simple and not described in an experiment on high degree of freedom systems. The study of the application of adaptive controllers in dealing with master and slave model uncertainties, operator and environment force model uncertainties, unknown external disturbances, and communication delay was represented by V. Malyavej and A. V. Savkin [23], whose shortcomings were that the influence of other hardware, such as sensor performance, on the experiment is not described. For problems of system control and state estimation, the method of a state estimation for a continuous-time uncertain system via a digital communication channel with bit-rate constraints was proposed, in which optimal and suboptimal recursive coder–decoder state estimation schemes [24] were considered, but it was limited to hardware facilities to verify the proposed method.

In view of the above problems, thus, the method of dealing with system control and state estimation based on 6-DOF master and slave haptic manipulators is proposed. The study focuses on the requirements of stability, tracking, and transparency of an asymmetric teleoperation system. An asymmetric teleoperation system was designed; a 6-DOF haptic device was designed as the master-robot, and a dual-arm robot was chosen as the slave-robot. Aiming at the tracking and transparency of the asymmetric teleoperation system, a robust control algorithm based on the state feedback observer was proposed. The application of a robust controller in a robot teleoperation system to deal with master–slave model uncertainty, operator and environmental force model uncertainty, unknown external interference, and communication delay is discussed, respectively, and finally, the correctness and effectiveness of the algorithm is verified by hardware experiments. The stability, tracking, and transparency of the system were verified based on simulation experiments. Experiments of tracking and transparency were carried out on the real asymmetric teleoperation system, and the effectiveness of the robust control algorithm was verified.

The paper is organized as follows: Section 2 provides the structural design of the haptic device and the asymmetric teleoperation system. The simulation experiment of the PID control algorithm is given in Section 3. A robust control algorithm is proposed and experimental tests are carried out in Section 4. Section 5 is results and discussion. Finally, the conclusions are presented in Section 6.

## 2. Materials and Methods

### 2.1. Positioning Mechanism Designation of the New Haptic Device

The structure of robots can be divided into series: parallel and hybrid. A series mechanism connects the operation end with the base through multiple connecting rods, which belongs to an open chain structure. A parallel mechanism contains at least two independent kinematic chains between the end and the base. The third is hybrid; in this paper, a hybrid structure of series parallel connection is adopted to design the mechanism of a haptic device. It has two parts: the positioning mechanism and directional mechanism, and it is a 6-DOF hybrid haptic device.

Generally speaking, the workspace of a haptic device is smaller than that of a virtual simulation environment; therefore, when mapping the operation space. If the workspace of the master and the slave is inconsistent, problems arise in the mapping of the displacement scale factor, reachable workspace, and direction of movement or rotation.

**Figure 2:**
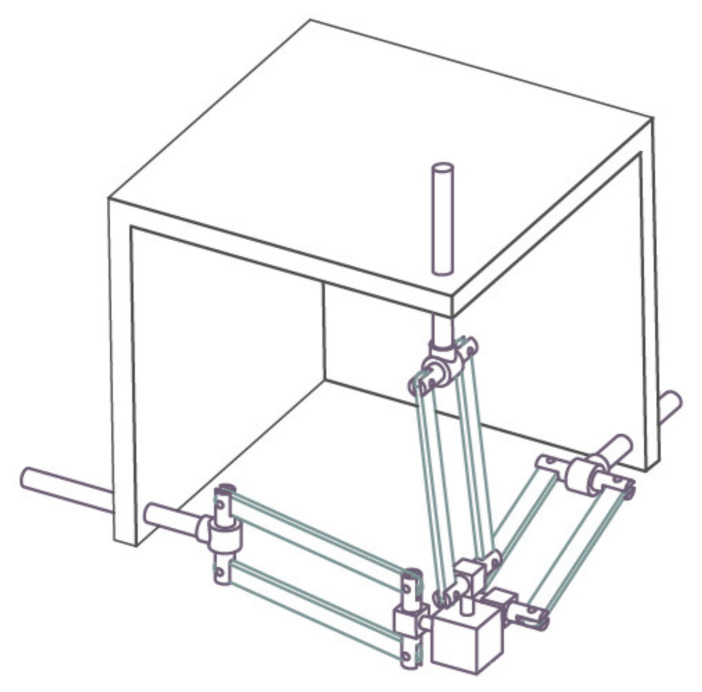
3PRPaR mechanism diagram.

In order to eliminate these problems, the workspace of the haptic device should be a regular cube space, which can correspond to the actual or virtual three-dimensional space. At the same time, the general application needs to keep the whole mechanism as compact as possible. In addition, to achieve fast motion, the moving parts of the master hand should have small inertia. An improved translational mechanism 3PRPaR was described [25] (Figure 2).

**Figure 3:**
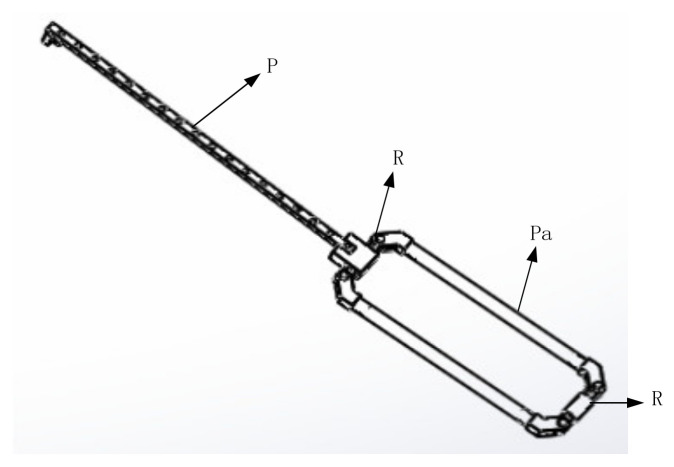
PRPaR branch chain.

**Figure 4:**
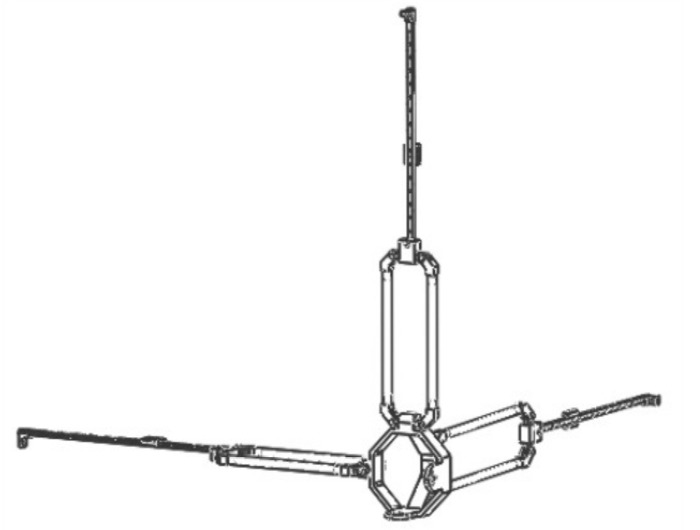
3PRPaR positioning mechanism structure.

The direction of the moving pair of the mechanism is consistent with the direction of the rod length; that is, the direction of force transmission is also along the direction of the rod length, so it has high structural stiffness [26]. It can achieve a large output of feedback force. Due to the vertical installation of the moving joint, it has an isotropic configuration in its workspace. In addition, the workspace of the mechanism is approximately a regular cube, which is convenient for master–slave motion mapping. Therefore, the mechanism is more suitable as a haptic device [27]. The schematic diagram of the branch chain structure and three branch chain combination structure are shown in Figure 3 and Figure 4. The branch chain of 3PRPaR can move along x, y, and z directions, so the positioning mechanism has three degrees of freedom.

### 2.2. Directional Mechanism Designation of the New Haptic Device

**Figure 5:**
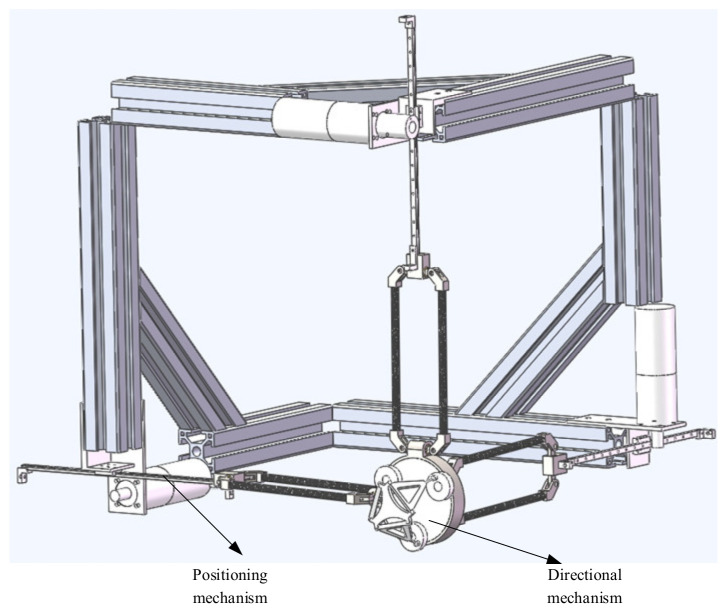
Overall assembly diagram of the haptic device.

In 1998, German professor Joachim Lueckel designed a 6-DOF parallel robot Triplanar. The design conception of ROBO_003 comes from triplanar, and it also has its own obvious characteristics [28]. There are 3 2-DOF planar motors in the parallel robot Triplanar; the 3 motors are replaced by three turntables that rotate around a fixed axis in ROBO_003. It is equivalent to limiting the two degree of freedom motion of three plane motors to the single degree of freedom along the fixed circle. The parallel robot ROBO_ 003 is a 3-DOF pure rotation mechanism, which is along the x, y, z axes, respectively. The overall assembly diagram of the haptic device is shown in Figure 5.

### 2.3. Designation of a New Asymmetric Teleoperation System

**Figure 6:**
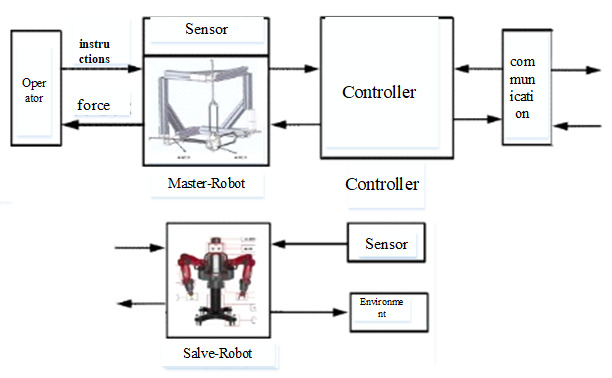
Asymmetric Teleoperation System.

A complete teleoperation system is composed of an operator, master robot, communication, slave robot, and the environment. The new asymmetric teleoperation system designed in this paper is divided into a master control system and slave control system; the master control system consists of a six degrees of freedom haptic device, a controller, and communication. The new asymmetric teleoperation system is shown in Figure 6.

## 3. Tracking Performance of the Asymmetric Teleoperation System

### 3.1. Dynamics of Master-Slave Robot

**Figure 7:**
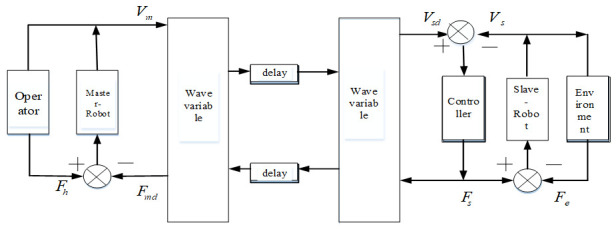
Control structure of a multi-DOF teleoperation system.

The control structure of the multi-DOF teleoperation system is shown in Figure 7. It is composed of five parts: an operator, master robot, communication channel, slave robot, and the environment [29]. The mathematical model of a multi-DOF teleoperation system can be obtained by analyzing the mathematical model of each part.

The dynamics of the master–slave robot teleoperation system is described as follows:

(1) $$\begin{array}{l} M_{m}(q_{m}){\ddot{q}}_{m}+C_{m}(q_{m},{\dot{q}}_{m}){\dot{q}}_{m}+g_{m}(q_{m})=\tau_{m}+J_{m}^{T}(q_{m})F_{h}\\ M_{s}(q_{s}){\ddot{q}}_{s}+C_{s}(q_{s},{\dot{q}}_{s}){\dot{q}}_{s}+g_{s}(q_{s})=\tau_{s}-J_{s}^{T}(q_{s})F_{e} \end{array}$$

m and s represent the master robot and the slave robot, respectively. h and e represent the operator and the environment, respectively. For any j∈{m,s}, $q_{j},{\dot{q}}_{j},{\ddot{q}}_{j}$ represent the joint position, velocity, and acceleration of the robot, respectively. $M_{j}(q_{j})$ is the inertial matrix of the robot. $C_{j}(q_{j},{\dot{q}}_{j})$ is centrifugal force and Coriolis force matrix. $g_{j}(q_{j})$ is gravity vector, $\tau_{j}$ is torque, and $F_{h}$ and $F_{e}$ represent the torque which is applied to the master robot by the operator and the torque exerted by the environment on the slave robot, respectively. $x_{j}$ represents position and pose coordinates of the robot end effector in the workspace. It is defined as:

(2) $$x_{j}=h_{j}(q_{j}),\;{\dot{x}}_{j}=\frac{\partial h_{j}(q_{j})}{\partial q_{j}}{\dot{q}}_{j}=J_{j}(q_{j}){\dot{q}}_{j}$$

The parameters of each rod of the dual-arm robot are shown in Table 1.

The end effector of the slave robot can be regarded as a mass-spring-damping model. Its dynamic model is regarded as a two-order system [30]:

$\frac{1}{m_{h}s^{2}+b_{h}s+k_{h}}$, $m_{h},b_{h},k_{h}$ represents mass, damping, and elastic coefficient of operator’s arm, respectively. The environment is directly in contact with the manipulator and can be modeled as a mass damping elastic model; it is assumed that the end effector of the slave robot is always in contact with the environment during operation. Therefore, the displacement of the environment is equal to the displacement from the manipulator $x_{s}$. Ignoring the unknown factors in the environment, the dynamic model of the environment is as follows:

$F_{e}=(m_{e}s^{2}+b_{e}s+k_{e})x_{s}$, $m_{e},b_{e},k_{e}$ represents the mass, damping, and elastic coefficient of environment, respectively, $F_{s}$ is the force applied from the end effector to the environment, $F_{s}=-F_{e}$.

### 3.2. Trajectory Tracking Experiment of Slave Robot End Effector

The end effector trajectory of the haptic device is tracked by the end effector of the slave robot. Using single joint control to achieve the trajectory tracking, let the tracking error of the force be: $e_{f}=F_{m}-F_{h}$, and $F_{m}$ is force applied on the haptic device. The PID control algorithm is:

(3) $$\tau_{m}=k_{p}e_{f}+k_{i}\int e_{f}dt+k_{d}{\dot{e}}_{f}$$

**Figure 8:**
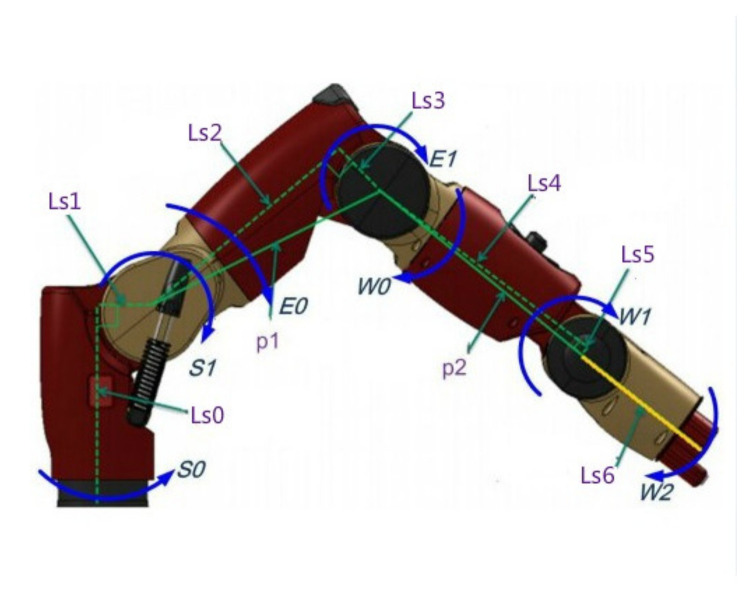
Baxter’s right arm.

**Figure 9:**
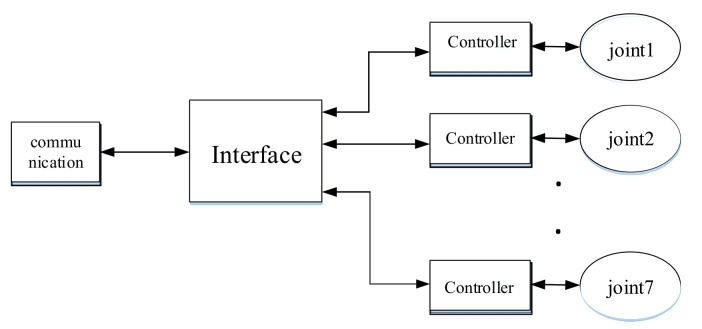
System of PID single-joint control.

PID setting parameters of each rod of the right arm are shown in Table 2:

Baxter’s right arm and the system of PID single-joint control are shown in Figure 8 and Figure 9.

### 3.3. Simulation of PID Control Algorithm

**Figure 10:**
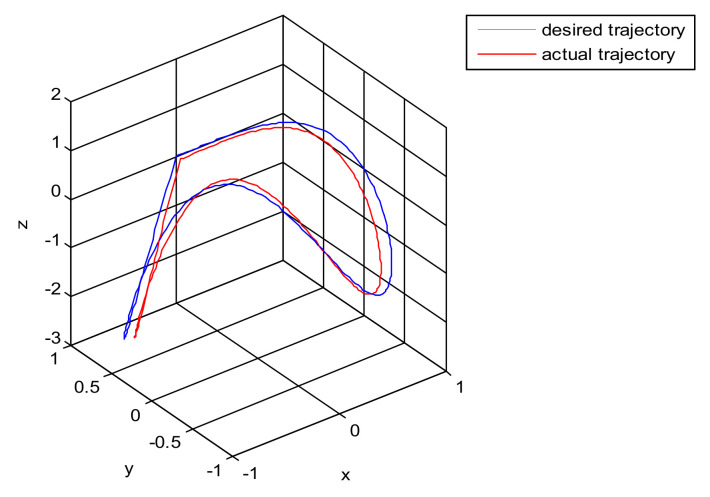
The trajectory tracking of end effector of Baxter right arm.

The simulation of the asymmetric teleoperation robot in this paper is based on a platform. In this paper, each subsystem module in the system was established as an S-function module. The simulation model of the teleoperation system is constituted by connecting the modules in order. The square root sum of the tracking errors in each direction defines the end tracking error. The expected trajectories in three directions, respectively, are: x = sin(t); y = cos(t); z = t · sin(t) · cos(t). The end effector trajectory of the right arm is shown in Figure 10. The red line represents the actual trajectory, and the blue line is desired trajectory. The maximum displacement trajectory error along x, y, and z directions of the end effector is 0.1, 0.045, and 0.028, respectively. It can be concluded that the PID control algorithm does not satisfy the requirement of the teleoperation system as shown in Figure 10. It is necessary to design a new controller for the teleoperation system.

### 3.4. Sliding Mode Control Algorithm

Using sliding mode control to achieve the trajectory tracking, let the tracking error of the force be: $e_{f}=F_{m}-F_{h}$, and $F_{m}$ is the force applied on the haptic device. The sliding mode control algorithm is:

(4) $$e=x_{m}-x_{s},\;\dot{e}={\dot{x}}_{m}-{\dot{x}}_{s},\;\ddot{e}={\ddot{x}}_{m}-{\ddot{x}}_{s}$$

The sliding mode function is:

(5) $$\begin{array}{l} s=ce+\dot{e},c>0\\ \dot{s}=c\dot{e}+\ddot{e}=c\dot{e}+{\ddot{x}}_{m}-(u_{s}+d_{s}-b_{s}{\dot{x}}_{s}-c_{w}x_{s})/M_{s} \end{array}$$

The sliding mode control law is:

(6) $$u_{s}=M_{s}(c\dot{e}+{\ddot{x}}_{m})+b_{s}{\dot{x}}_{s}+c_{w}x_{s}+(D_{s}+\eta )\mathrm{sgn}s$$

(7) $$\mathrm{Therefore}:\;s\dot{s}=-\frac{(D_{s}+\eta )\left|s\right|+d_{s}s}{M_{s}}\leq -\frac{\eta }{M_{s}}\left|s\right|$$

### 3.5. Simulation of Sliding Mode Control Algorithm

**Figure 11:**
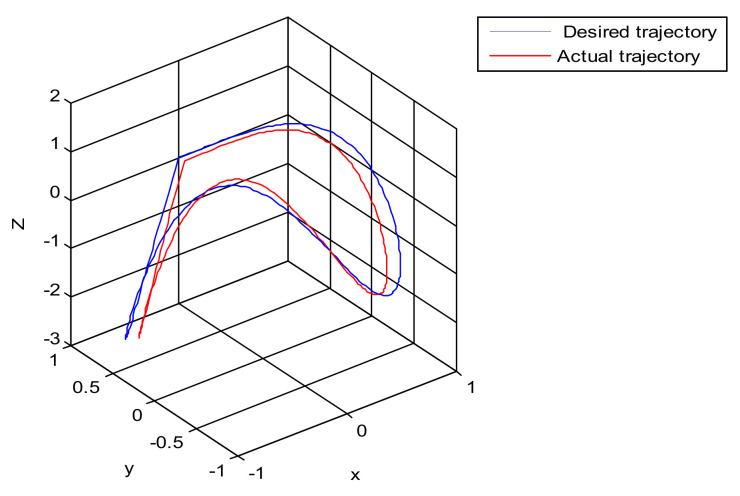
The trajectory tracking of end effector of Baxter right arm (sliding mode control).

**Figure 12:**
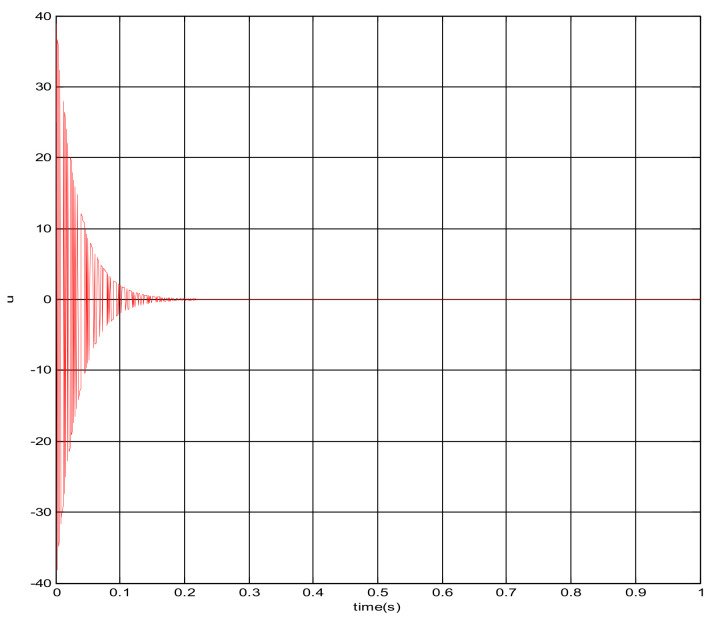
The changing curve of control quantity u.

The square root sum of the tracking errors in each direction defines the end tracking error. The expected trajectories in three directions, respectively, are: x = sin(t); y = cos(t); z = t · sin(t) · cos(t). The end line represents the actual trajectory and the blue line is desired trajectory. It can be concluded that the sliding mode control algorithm does not satisfy the requirement of the teleoperation system as shown in Figure 11. The control quantity u has a chattering phenomenon which is shown in Figure 12.

## 4. Robust Control Algorithm of the Asymmetric Teleoperation System Based on State Observer

The PID control algorithm in the master–slave robot teleoperation system is unsatisfactory, as many factors are ignored, such as interference, modeling error, and so on [31]. The accuracy, tracking, and transparency of the system in the actual control cannot meet the expectations. After adding the model error to the original system dynamics equation, the dynamics of the master–slave robot teleoperation system is described as follows:

(8) $$\begin{array}{l} M_{m}(q_{m}){\ddot{q}}_{m}+C_{m}(q_{m},{\dot{q}}_{m}){\dot{q}}_{m}+g_{m}(q_{m})+\xi_{m}(q_{m},{\dot{q}}_{m})=\tau_{m}+J_{m}^{T}(q_{m})F_{h}=\tau_{m}+F_{m}\\ M_{s}(q_{s}){\ddot{q}}_{s}+C_{s}(q_{s},{\dot{q}}_{s}){\dot{q}}_{s}+g_{s}(q_{s})+\xi_{s}(q_{s},{\dot{q}}_{s})=\tau_{s}-J_{s}^{T}(q_{s})F_{e}=\tau_{s}-F_{s}\\ F_{e}=(m_{e}s^{2}+b_{e}s+k_{e})x_{s}+\xi_{0}(q_{s},{\dot{q}}_{s}) \end{array}$$

$\xi_{m}(q_{m},{\dot{q}}_{m}),\xi_{s}(q_{s},{\dot{q}}_{s})$ represent the model error of the master–slave robot, respectively. $\xi_{0}(q_{s},{\dot{q}}_{s})$ is the model error representing the reaction force of the object being grasped; it is a nonlinear function [32]. Let $\xi_{m},\xi_{s},\xi_{0}$ be unknown, and their gains are bounded. There are constants $\sigma_{m1},\sigma_{m2},\sigma_{s1},\sigma_{s2}$ and $\sigma_{w1},\sigma_{w2}$, making the following formula true for any $x_{m},{\dot{x}}_{m},x_{s},{\dot{x}}_{s}$.

(9) $$\begin{array}{l} \xi_{m}^{2}({\dot{x}}_{m},x_{m})\leq \sigma_{m1}^{2}{\dot{x}}_{m}^{2}+\sigma_{m2}^{2}x_{m}^{2}\\ \xi_{s}^{2}({\dot{x}}_{s},x_{s})\leq \sigma_{s1}^{2}{\dot{x}}_{s}^{2}+\sigma_{s2}^{2}x_{s}\\ \xi_{0}^{2}({\dot{x}}_{s1},x_{s})\leq \sigma_{w1}^{2}{\dot{x}}_{s}^{2}+\sigma_{w2}^{2}x_{s} \end{array}$$

Obviously, the bigger $\sigma_{mi}$ is, the larger the error of the master–slave mathematical model. The larger the uncertainty range of the captured object is, the bigger $\sigma_{w_{1}},\sigma_{w_{2}}$ are [33]. Let state variable $x^{T}=[q_{m}\;{\dot{q}}_{m}\;q_{s}\;{\dot{q}}_{s}]$, vector $u^{T}=[\tau_{m}\;\tau_{s}]$ is the input vector for control. The dynamic equation of the master–slave robot can be expressed as follows:

(10) $$\begin{array}{l} \dot{x}=Ax+B_{1}F+B_{2}u+\varphi (q)+\phi (q)\\ F=[F_{m}\;F_{s}]\;\;u=[\tau_{m}\;\tau_{s}] \end{array}\;A=\left[\begin{array}{cccc} 0 & 1 & 0 & 0\\ 0 & -\frac{C_{m}}{M_{m}} & 0 & 0\\ 0 & 0 & 0 & 1\\ 0 & 0 & 0 & -\frac{C_{s}}{M_{s}} \end{array}\right],\;B_{1}=\left[\begin{array}{cc} 0 & 0\\ \frac{1}{M_{m}} & 0\\ 0 & 0\\ 0 & -\frac{1}{M_{s}} \end{array}\right],\;B_{2}=\left[\begin{array}{cc} 0 & 0\\ \frac{1}{M_{m}} & 0\\ 0 & 0\\ 0 & \frac{1}{M_{s}} \end{array}\right],\;\varphi (q)=\left[\begin{array}{c} 0\\ -\frac{1}{M_{m}}\xi_{m}(q_{m},{\dot{q}}_{m})\\ 0\\ -\frac{1}{M_{s}}\xi_{s}(q_{s},{\dot{q}}_{s}) \end{array}\right],\;\phi (q)=\left[\begin{array}{c} 0\\ -\frac{g_{m}(q_{m})}{M_{m}}\\ 0\\ -\frac{g_{s}(q_{s})}{M_{s}} \end{array}\right]$$

where $F_{h}=0$, u=0, x=0 is the equilibrium point in Equation (6). When $F_{h}\neq 0$, the operator grasps the master robot so that one may feel that the master robot can directly grasp the object or encounter obstacles, improving the transparency of the master–slave teleoperation system [34].

Because the force $F_{h}$ produced by the operator is usually not a regular signal, we consider an $L_{2}$ gain method by reducing the force $F_{h}$ from the operator to the tracking error signal, to suppress the position and force errors caused by the gripping force. Two definition matrices $E_{1},E_{2}$ are as follows:

(11) $$E_{1}=\left[\begin{array}{ccc} 0 & 0 & 0\\ -\frac{1}{M_{m}} & 0 & 0\\ 0 & 0 & 0\\ 0 & -\frac{1}{M_{s}} & -\frac{1}{M_{s}} \end{array}\right],\;E_{2}=\left[\begin{array}{ccc} 0 & 0 & 0\\ 0 & 0 & -q_{2}\\ 0 & 0 & 0\\ 0 & 0 & 0 \end{array}\right]$$

The unknown function vector φ(q) and ψ(x) can be expressed as:

(12) $$\left[\begin{array}{c} \varphi (q)\\ \psi (x) \end{array}\right]=\left[\begin{array}{c} E_{1}\\ E_{2} \end{array}\right]\Sigma (x)$$

The unknown vector function Σ(x) is defined as:

(13) $$\Sigma (x)=\left[\begin{array}{c} \xi_{m}(q_{m},{\dot{q}}_{m})\\ \xi_{s}(q_{s},{\dot{q}}_{s})\\ \xi_{0}(q_{s},{\dot{q}}_{s}) \end{array}\right]$$

According to the hypothesis, Σ(x) is gain bounded. Define a constant matrix as:

(14) $$F=\left[\begin{array}{cccc} 0 & \sigma_{m_{1}} & 0 & 0\\ \sigma_{m_{2}} & 0 & 0 & 0\\ 0 & 0 & 0 & \sigma_{s_{1}}\\ 0 & 0 & \sigma_{s_{2}} & 0\\ 0 & 0 & 0 & \sigma_{w_{1}}\\ 0 & 0 & \sigma_{w_{2}} & 0 \end{array}\right],\;\left\|\Sigma (x)\right\|\leq \left\|Fx\right\|,\forall x$$

Therefore, the designation problem of the master–slave teleoperation control system is as follows, for the given controlled object with uncertainty:

(15) $$\left\{\begin{array}{l} \dot{x}=Ax+B_{1}F+B_{2}u+\varphi (q)+\phi (q)\\ F=[F_{m}\;F_{s}]\;\;u=[\tau_{m}\;\tau_{s}]\\ z=C_{1}x+D_{1}F_{h}+D_{2}u+\psi (x) \end{array}\right.$$

Seeking $u=K_{1}x+K_{2}F$, for any $\left\|\Sigma (x)\right\|\leq \left\|Fx\right\|,\forall x$ and unknown function Σ(x), the closed loop system satisfies:

(S1) when F=0, x=0 is equilibrium point of asymptotically stable closed loop system.
(S2) when x(0)=0, for any given T>0, ${\int_{0}^{T}\left\|z\right\|}^{2}dt\leq {\int_{0}^{T}\left\|F\right\|}^{2}dt,\forall F$ is true.

The closed loop system is:

(16) $$\begin{array}{c} \dot{x}=A_{k}x+B_{k}F+E_{1}\Sigma (x)\\ z=C_{k}x+D_{k}F+E_{2}\Sigma (x) \end{array}\;\begin{array}{c} A_{k}=A+B_{2}K_{1}\\ B_{k}=B_{1}+B_{2}K_{2} \end{array},\;\begin{array}{c} C_{k}=C_{1}+D_{2}K_{1}\\ D_{k}=D_{1}+D_{2}K_{2} \end{array}$$

For the designation of the ideal controller, define the auxiliary control object as:

(17) $$\begin{array}{l} \dot{x}=Ax+{\hat{B}}_{1}w+B_{2}v\\ \mu ={\hat{C}}_{1}x+{\hat{D}}_{1}w+{\hat{D}}_{2}v \end{array}\;{\hat{B}}_{1}=[B_{1}\;E_{1}],{\hat{C}}_{1}=\left[\begin{array}{c} C_{1}\\ f \end{array}\right],\;{\hat{D}}_{1}=\left[\begin{array}{cc} D_{1} & E_{2}\\ 0 & 0 \end{array}\right],{\hat{D}}_{2}=\left[\begin{array}{c} D_{2}\\ 0 \end{array}\right]$$

Set the auxiliary input signal as:

(18) $$w=\left[\begin{array}{c} F\\ \Sigma (x) \end{array}\right],z=[I\;\;0]\mu$$

The feedback control vector is: $v=K_{1}x+[K_{2}\;0]w$. The close-loop system is:

$$\begin{array}{l} \dot{x}=Ax+{\hat{B}}_{1}w+B_{2}v\\ \mu ={\hat{C}}_{1}x+{\hat{D}}_{1}w+{\hat{D}}_{2}v \end{array}$$

Additionally, $v=K_{1}x+[K_{2}\;0]w$ is equivalent to the system:

(19) $$\begin{array}{l} \dot{x}=A_{k}x+B_{k}F+E_{1}\Sigma (x)\\ z=C_{k}x+D_{k}F+E_{2}\Sigma (x) \end{array}$$

Closed-loop transfer function from w to μ is:

(20) $$T_{\mu w}(s)={\hat{D}}_{K}+{\hat{C}}_{K}{\left(sI-A_{K}\right)}^{-1}{\hat{B}}_{K}\;{\hat{B}}_{K}={\hat{B}}_{1}+B_{2}[K_{2}\;0],\;{\hat{C}}_{K}={\hat{C}}_{1}+{\hat{D}}_{2}[K_{2}\;0],\;{\hat{D}}_{K}={\hat{D}}_{1}+{\hat{D}}_{2}[K_{2}\;0]$$

Obviously, $T_{\mu w}(s)$ is a 4-order complex function matrix; define the calibration coefficient matrix as:

(21) $$\Lambda =\left[\begin{array}{cccc} \lambda_{1} & 0 & 0 & 0\\ 0 & \lambda_{2} & 0 & 0\\ 0 & 0 & \lambda_{3} & 0\\ 0 & 0 & 0 & \lambda_{4} \end{array}\right],\lambda_{i}>0,(i=1,2,3,4)$$

### 4.1. Stability Proof of State Feedback Control Algorithm

The transfer function of a close-loop system is assumed to be strictly analytic in the S-closed right-half plane. If there is an appropriate calibration function $\lambda_{i}>0,(i=1,2,3,4)$, make ${\left\|\Lambda^{-1}T_{\mu w}(s)\Lambda \right\|}_{\infty }<1$ be true, then the close-loop system satisfies the design requirements (S1),(S2). The output of the close-loop system is $\mu =T_{\mu w}(s)w$, multiply the two sides of the above formula by the inverse matrix of the calibration coefficient matrix, and obtain:

(22) $$\Lambda^{-1}\mu =\Lambda^{-1}T_{\mu w}(s)\Lambda \Lambda^{-1}w$$

Define the auxiliary input and output signal: $\hat{\mu }=\Lambda^{-1}\mu$, $\hat{w}=\Lambda^{-1}w$, ${\left\|\Lambda^{-1}T_{\mu w}(s)\Lambda \right\|}_{\infty }<1$, meaning that the $L_{2}$ gain of the close-loop system from $\hat{w}$ to $\hat{\mu }$ is strictly less than 1, that is:

(23) $${\left\|\hat{\mu }\right\|}_{2}<{\left\|\hat{w}\right\|}_{2},\forall \hat{w}$$

The existence of the Lyapunov function V(x) along the state trajectory of the system satisfies the time differential [35]:

(24) $$\dot{V}<\frac{1}{2}\{{\left\|\hat{w}\right\|}^{2}-{\left\|\hat{\mu }\right\|}^{2}\}\;\forall \hat{w},\;\hat{w}=\Lambda^{-1}\left[\begin{array}{c} F\\ \Sigma (x) \end{array}\right]$$

(25) $$\hat{\mu }=\Lambda^{-1}\mu =\Lambda^{-1}\left[\begin{array}{c} z\\ Fx \end{array}\right],\;\Lambda =\left[\begin{array}{cc} \lambda_{1} & 0\\ 0 & \lambda_{2} \end{array}\right],\;\Lambda_{0}=\left[\begin{array}{ccc} \lambda_{2} & 0 & 0\\ 0 & \lambda_{3} & 0\\ 0 & 0 & \lambda_{4} \end{array}\right]$$

So $\dot{V}<\frac{1}{2}\{{\left\|\hat{w}\right\|}^{2}-{\left\|\hat{\mu }\right\|}^{2}\}\;\forall \hat{w}$ is equivalent to:

(26) $$\dot{V}<\frac{1}{2}\{\frac{1}{\lambda_{1}^{2}}{\left\|F\right\|}^{2}-\frac{1}{\lambda_{1}^{2}}{\left\|z\right\|}^{2}\}\;+\frac{1}{2}\{{\left\|\Lambda_{0}^{-1}\Sigma (x)\right\|}^{2}-{\left\|\Lambda_{0}^{-1}fx\right\|}^{2}\}$$

Let $f=\left[\begin{array}{c} f_{1}\\ f_{2}\\ f_{3} \end{array}\right]$, $f_{i}(i=1,2,3)$ are a 2 * 4 matrix, according to $\begin{array}{l} \xi_{m}^{2}({\dot{x}}_{m},x_{m})\leq \sigma_{m1}^{2}{\dot{x}}_{m}^{2}+\sigma_{m2}^{2}x_{m}^{2}\\ \xi_{s}^{2}({\dot{x}}_{s},x_{s})\leq \sigma_{s1}^{2}{\dot{x}}_{s}^{2}+\sigma_{s2}^{2}x_{s}\\ \xi_{0}^{2}({\dot{x}}_{s1},x_{s})\leq \sigma_{w1}^{2}{\dot{x}}_{s}^{2}+\sigma_{w2}^{2}x_{s} \end{array}$,

Obtain:

(27) $${\left\|\xi_{m}\right\|}^{2}\leq {\left\|f_{1}x\right\|}^{2},{\left\|\xi_{s}\right\|}^{2}\leq {\left\|f_{2}x\right\|}^{2},{\left\|\xi_{0}\right\|}^{2}\leq {\left\|f_{3}x\right\|}^{2}$$

$\dot{V}<\frac{1}{2}\{\frac{1}{\lambda_{1}^{2}}{\left\|F\right\|}^{2}-\frac{1}{\lambda_{1}^{2}}{\left\|z\right\|}^{2}\}\;+\frac{1}{2}\{{\left\|\Lambda_{0}^{-1}\Sigma (x)\right\|}^{2}-{\left\|\Lambda_{0}^{-1}fx\right\|}^{2}\}$ can be expressed as:

(28) $$\dot{V}<\frac{1}{2\lambda_{1}^{2}}\{{\left\|F\right\|}^{2}-{\left\|z\right\|}^{2}\}+\frac{1}{2\lambda_{2}^{2}}\{{\left\|\xi_{m}({\dot{x}}_{m},x_{m})\right\|}^{2}-{\left\|f_{1}x\right\|}^{2}\}\;+\frac{1}{2\lambda_{3}^{2}}\{{\left\|\xi_{s}({\dot{x}}_{s},x_{s})\right\|}^{2}-{\left\|f_{2}x\right\|}^{2}\}+\frac{1}{2\lambda_{4}^{2}}\{{\left\|\xi_{0}({\dot{x}}_{s},x_{s})\right\|}^{2}-{\left\|f_{3}x\right\|}^{2}\}\leq \frac{1}{2\lambda_{1}}\{{\left\|F\right\|}^{2}-{\left\|z\right\|}^{2}\},\;\forall F$$

Let the initial state be zero, note that V(x(0))=V(0)=0, and obtain:

(29) $$\int_{0}^{T}{\left\|z\right\|}^{2}+2\lambda_{1}V(x(T))<{\int_{0}^{T}\left\|F\right\|}^{2}dt,\;\forall F$$

The close-loop system: $\begin{array}{l} \dot{x}=A_{k}x+B_{k}F+E_{1}\Sigma (x)\\ z=C_{k}x+D_{k}F+E_{2}\Sigma (x) \end{array}$ meets the performance requirements S2 when F=0, $\dot{V}<\frac{1}{2\lambda_{1}}\{-{\left\|z\right\|}^{2}\}\;,\forall F<0$, meets the performance requirements S1. That is, the Lyapunov function V(x) can guarantee the robust stability of the close-loop system. If we can get vector $v=K_{1}x+[K_{2}\;0]w$, which makes ${\left\|\Lambda^{-1}T_{\mu w}(s)\Lambda \right\|}_{\infty }<1$ true. Therefore, the designation problem of the robust feedback control system for a master–slave robot is the problem of the auxiliary nominal system.

### 4.2. Designation of State Observer

If the speed of the end effector from the slave robot is not measurable, the state observer can be considered. However, due to the nonlinear uncertainty of the plant and that the close-loop system must satisfy the $L_{2}$ gain, the observer cannot be designed based on the separation principle [36]. We re-examine the performance of the observer based on the close-loop system. The position of the end effector is assumed to be measurable; that is:

(30) $$y=C_{2}x,\;C_{2}=\left[\begin{array}{cccc} 1 & 0 & 0 & 0\\ 0 & 0 & 1 & 0 \end{array}\right]$$

State that feedback control vector $u=K_{1}x+K_{2}F$ is set by formula ${\left\|\Lambda^{-1}T_{\mu w}(s)\Lambda \right\|}_{\infty }<1$; an observer-based controller with the following structure is considered:

(31) $$\begin{array}{l} u=K_{1}\hat{x}+K_{2}F\\ \dot{\hat{x}}=A\hat{x}+B_{1}F+B_{2}u+L(y-C_{2}\hat{x}) \end{array}$$

$\hat{x}$ is the estimated value of x, L is the gain of the observer to be designed, and $K_{1},K_{2}$ is the feedback and feedforward gain of the robust control vector. Let $\tilde{D}=\Lambda^{-1}{\hat{D}}_{k}\Lambda ,\tilde{C}=\Lambda^{-1}C_{k},\tilde{B}={\hat{B}}_{k}\Lambda$, according to $T_{\mu w}(s)={\hat{D}}_{K}+{\hat{C}}_{K}{\left(sI-A_{K}\right)}^{-1}{\hat{B}}_{K}$, obtain: $\Lambda^{-1}T_{\mu w}(s)\Lambda =\tilde{D}+\tilde{C}{\left(sI-A_{K}\right)}^{-1}\tilde{B}$. If ${\left\|\Lambda^{-1}T_{\mu w}(s)\Lambda \right\|}_{\infty }<1$ is true, according to the relationship between the $H_{\infty }$ norm of transfer function matrix and the Riccati equation: P>0, making the fellow Riccati Inequality true:

(32) $$A_{k}^{T}P+PA_{k}+{\tilde{C}}^{T}\tilde{C}+(P\tilde{B}+{\tilde{C}}^{T}\tilde{D}){\tilde{R}}^{-1}({\tilde{B}}^{T}P+{\tilde{D}}^{T}\tilde{C})<0,\;\tilde{R}=I-{\tilde{D}}^{T}\tilde{D}$$

The robust control algorithm of the observer-based master–slave teleoperation system is investigated experimentally; the master robot operation enables the slave robot to grasp objects with different elastic coefficients. The effect of the trajectory tracking and force feedback tracking of the master–slave under the new control algorithm is investigated. The parameters of the master robot and the captured object are as follows. The parameters of the slave robot are shown in Table 2.

$$M_{m}=\left[\begin{array}{cccccc} 0.385 & 0 & 0 & 0 & 0 & 0\\ 0 & 0.383 & 0 & 0 & 0 & 0\\ 0 & 0 & 0.383 & 0 & 0 & 0\\ 0 & 0 & 0 & 0.278 & 0 & 0\\ 0 & 0 & 0 & 0 & 0.276 & 0\\ 0 & 0 & 0 & 0 & 0 & 0.276 \end{array}\right],\;C_{m}=\left[\begin{array}{cccccc} 20.5 & 0 & 0 & 0 & 0 & 0\\ 0 & 18.2 & 0 & 0 & 0 & 0\\ 0 & 0 & 17.4 & 0 & 0 & 0\\ 0 & 0 & 0 & 16.3 & 0 & 0\\ 0 & 0 & 0 & 0 & 16.2 & 0\\ 0 & 0 & 0 & 0 & 0 & 16.2 \end{array}\right]$$

Setting uncertain parameters $m_{e}=0.045kg$$b_{e}=30N\cdot s/m$, $k_{e}=1500N/m$, $\sigma_{m1}=40,\sigma_{m2}=20$, $\sigma_{s1}=30,\sigma_{s2}=15$, $\sigma_{w1}=30,\sigma_{w2}=1000$. This means that the elastic coefficient of the object has a large range of uncertainty. The weighted coefficients describing the performance of interference suppression are: $q_{1}=5000,q_{2}=1,r_{1}=r_{2}=0.2$. The calibration coefficients are: $\lambda_{1}=0.5,\lambda_{2}=0.1,\lambda_{3}=0.5,\lambda_{4}=0.7$. To make $T_{\mu w}(s)={\hat{D}}_{K}+{\hat{C}}_{K}{\left(sI-A_{K}\right)}^{-1}{\hat{B}}_{K}$ strictly analytic in the S right half plane, the designation problems of $H_{\infty }$ have solutions. The values of feedback gain $K_{1}$ and feedforward gain $K_{2}$ which satisfies ${\left\|\Lambda^{-1}T_{\mu_{e}w}(s)\Lambda \right\|}_{\infty }<1$ are: $K_{1}=\left[\begin{array}{cccc} -21747 & -678.34 & 16276 & 57.352\\ 21624 & -60.389 & -23161 & -63.542 \end{array}\right]$, $K_{2}=\left[\begin{array}{c} 2.7450\\ 2.7450 \end{array}\right]$.

Given the gain matrix $K_{1},K_{2}$, and P>0, if there is a positive definite matrix Y and constant ε>0, make $YA_{M}^{T}+A_{M}Y+Y(Q-\frac{1}{\varepsilon }C_{2}^{T}C_{2})Y+S<0$ true, the close-loop system is:

(33) $$\dot{x}=Ax+B_{1}F+B_{2}u+\varphi (q)+\phi (q)\;z=C_{1}x+D_{1}F_{h}+D_{2}u+\psi (x)\;\begin{array}{l} u=K_{1}\hat{x}+K_{2}F\\ \dot{\hat{x}}=A\hat{x}+B_{1}F+B_{2}u+L(y-C_{2}\hat{x}) \end{array}$$

The observer’s gain which satisfies performance S1,S2 is given as follows:

(34) $$L=\frac{1}{2\varepsilon }YC_{2}^{T},\;\varepsilon =0.5$$

$YA_{M}^{T}+A_{M}Y+Y(Q-\frac{1}{\varepsilon }C_{2}^{T}C_{2})Y+S<0$ has positive definite solutions Y, calculate formula (34). Obtain the ideal observer gain:

$$L=\left[\begin{array}{cc} 1.0534 & 0.7023\\ -8.7831 & -10.2432\\ 0.8216 & 1.0399\\ 60.1712 & -107.01 \end{array}\right]$$

### 4.3. Trajectory Tracking Simulation Experiment of Robust Control Algorithm Based on State Observer

**Figure 13:**
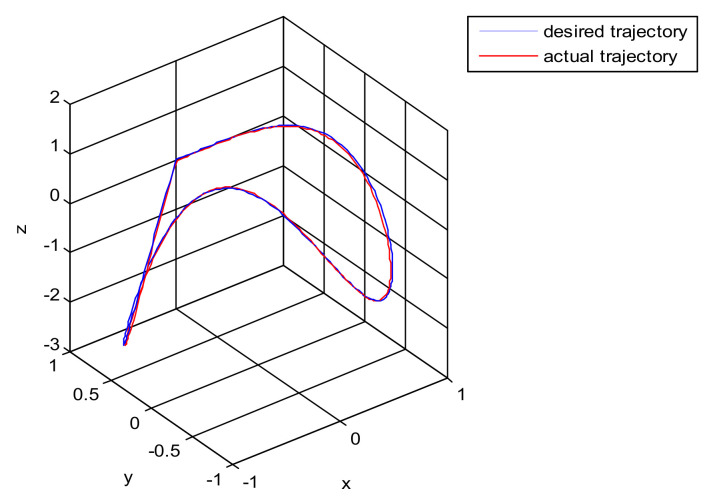
Trajectory tracking of end effector of Baxter’s right arm. (Simulation of robust control algorithm tracking based on state observer).

**Figure 14:**
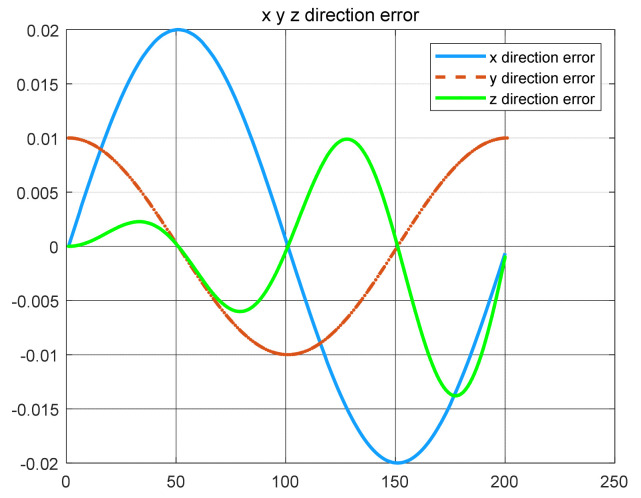
The trajectory tracking error of end effector, diagram of slave robot along x,y,z directions (robust control algorithm).

The robust control algorithm proposed was applied to the asymmetric teleoperation system. The operator manipulates the haptic device to make the slave robot move in the desired trajectory. Trajectory tracking of the end effector of Baxter’s right arm is shown in Figure 13. The square root sum of the trajectory tracking error in each direction defines the trajectory tracking error; the end effector trajectory tracking, and error of the right arm are shown in Figure 14. The red line represents actual trajectory, and the blue line represents desired trajectory. The maximum error of trajectory tracking in three directions are 0.02, 0.01, and 0.015, respectively. The trajectory tracking performance of the asymmetric teleoperation system is improved.

### 4.4. Object Grasping Experiment Simulation of Robust Control Algorithm Based on Observer

**Figure 15:**
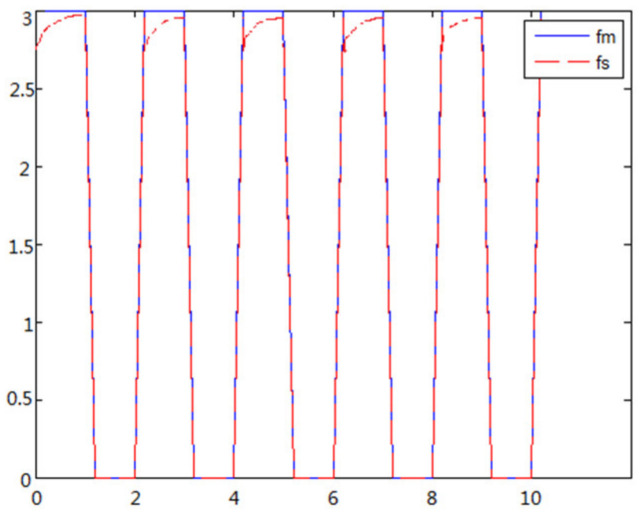
Force feedback along x axis. (No time delay).

**Figure 16:**
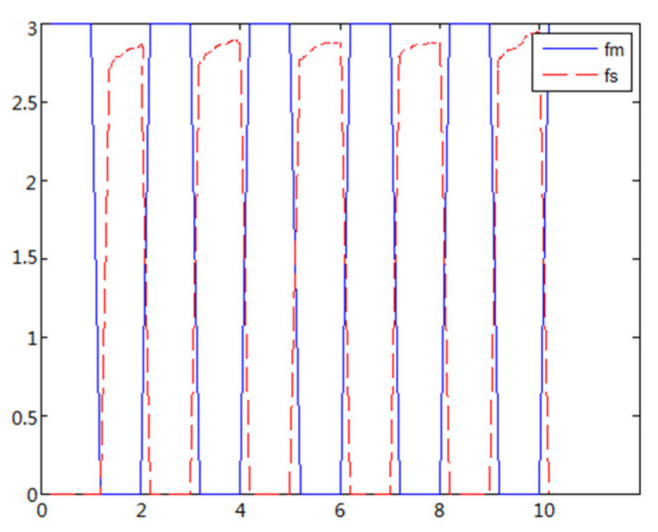
Force feedback along x axis. (Constant time delay).

**Figure 17:**
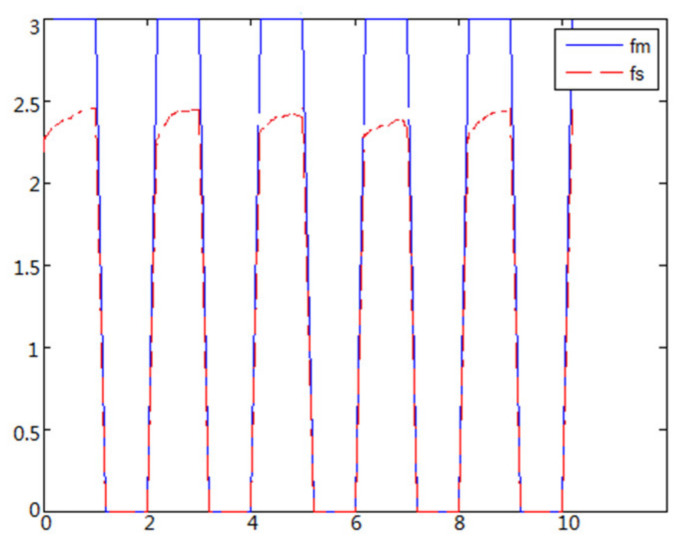
Force feedback along x axis. (No time delay, elastic ball).

**Figure 18:**
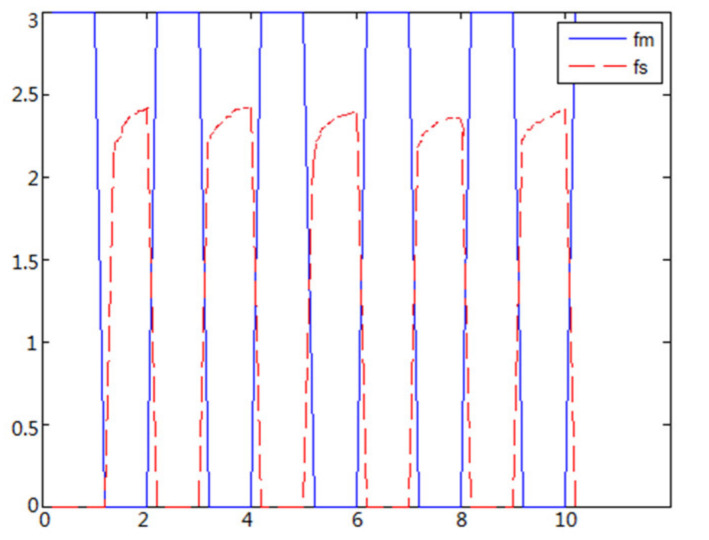
Force feedback along x axis. (Constant time delay, elastic ball).

(1) The object to be caught is a solid ball
  When the object is a solid ball, the elastic coefficient of the object is bigger, and parameter B is bigger. Actual force feedback along the x axis of the haptic device is shown in Figure 15 and Figure 16.
(2) The object to be caught is an elastic ball
  When the object is an elastic ball, the elastic coefficient of the object is smaller, and parameter B is smaller. Actual force feedback along the x axis of the haptic device is shown in Figure 17 and Figure 18.

### 4.5. Real Experiments on Real Asymmetric Teleoperation System

To test the tracking and transparency of the asymmetric teleoperation system, the visualization model of was built based on ROS. The track communication interface of Moveit was used and connected with the Gazebo simulation platform. Trajectory tracking and grasping experiments of the system was designed. Importing trajectory data from MATLAB, the experimental data of the robot in the gazebo simulation environment are obtained. Finally, the data obtained are analyzed. Two steps are started on ROS before the experiment.

**Figure 19:**
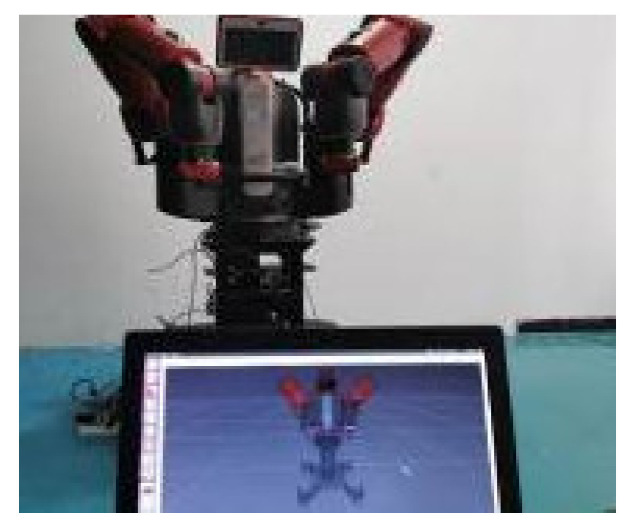
Original state.

**Figure 20:**
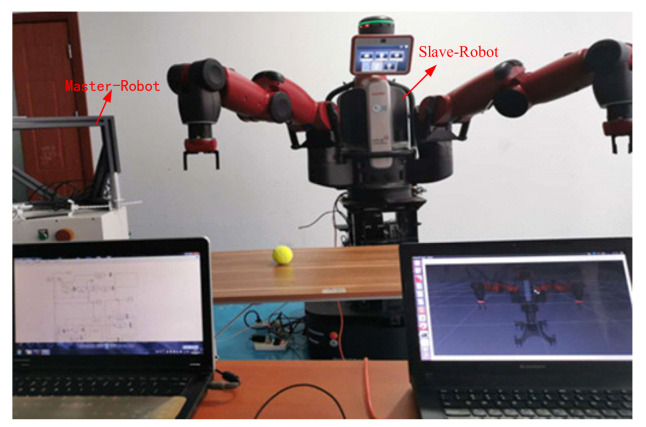
Experimental scene.

(1) Baxter Moveit! Configure. Start-up Rviz, the URDF model of Baxter was imported into system.
(2) Communication. Initialized Baxter, bring Baxter back to the original state, as shown in Figure 19. Experimental scene is shown in Figure 20.

#### 4.5.1. Real Trajectory Tracking Experiment on Asymmetric Teleoperation System

**Figure 21:**
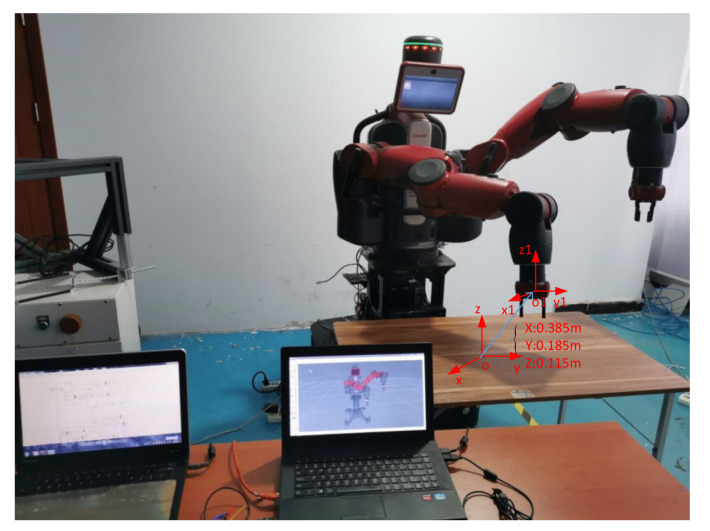
End effector displacements of Baxter’s right arm on the real system.

In the initial state, the delay is ignored. The end effector of the haptic device moves 0.01 m along directions separately. According to the operation space mapping algorithm, end effector displacements of Baxter’s right arm along directions are 0.385 m, 0.185 m, and 0.115 m separately, as shown in Figure 21.

#### 4.5.2. Transparency Experiment about Force Feedback on Real Asymmetric Teleoperation System

**Figure 22:**
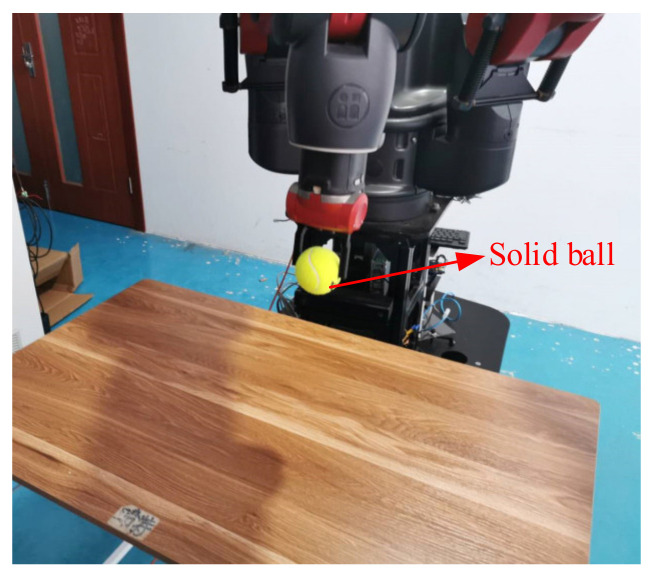
Grasping solid ball.

**Figure 23:**
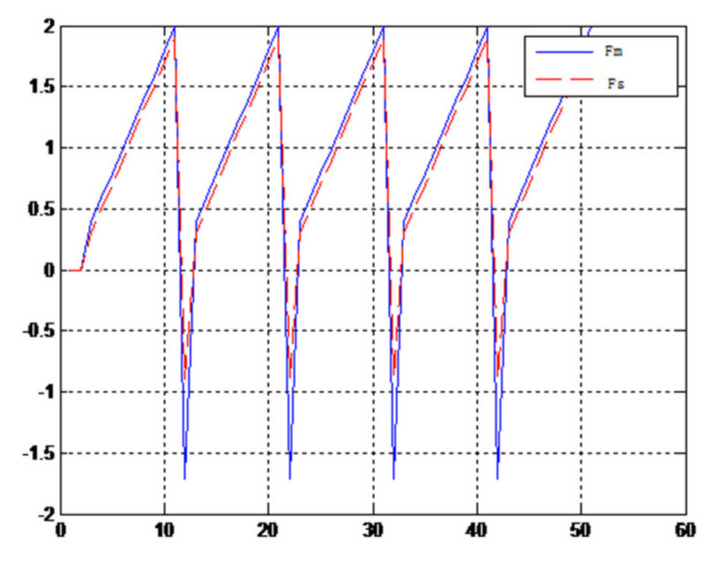
Force feedback wave along x axis. (No time delay, solid ball).

**Figure 24:**
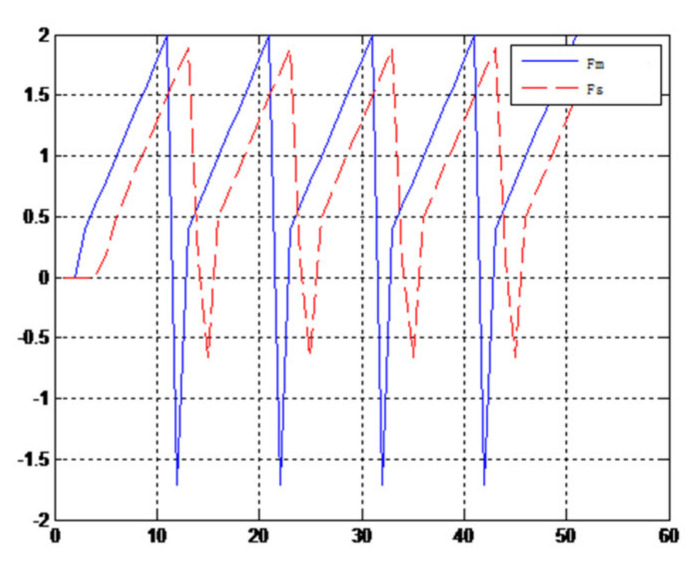
Force feedback wave along x axis. (Constant time delay, solid ball).

When the object is a solid ball, the elastic coefficient of the object is bigger, and the parameter of B is bigger. The interaction force can be measured by the force sensor which is fixed on the wrist. Then, it can be transferred to the master–robot by network. The solid yellow ball selected in this experiment is shown in Figure 22. Grasping the solid ball with the right arm is shown in Figure 22. The force feedback wave of the haptic device along the x axis when Baxter is grasping the solid ball is shown in Figure 23 and Figure 24 (robust control algorithm).

**Figure 25:**
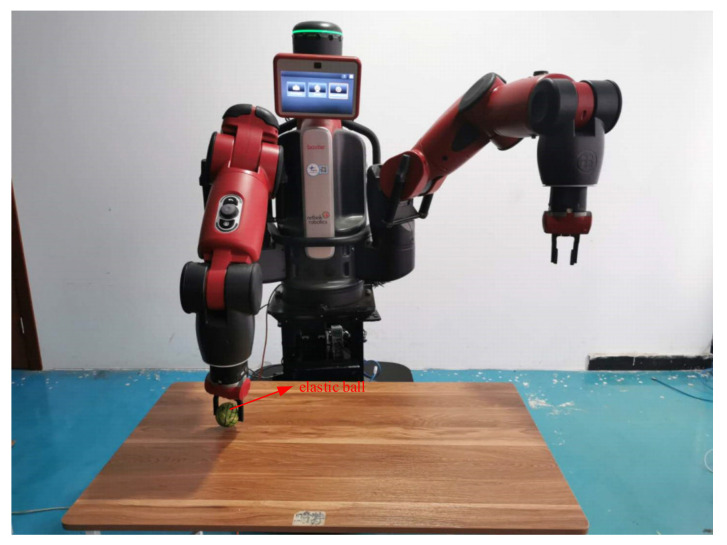
Grasping elastic ball.

**Figure 26:**
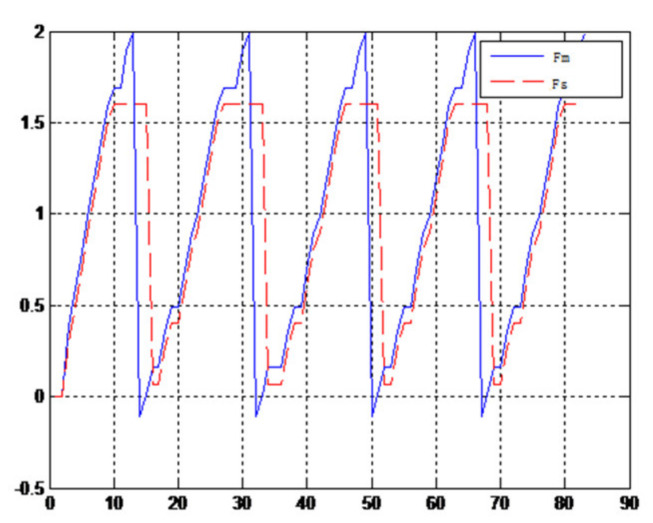
Force feedback wave along x axis. (No time delay, elastic ball).

**Figure 27:**
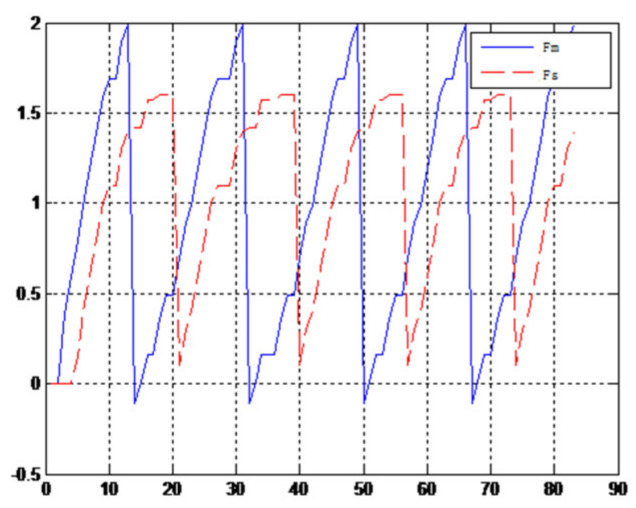
Force feedback wave along x axis. (Constant time delay, elastic ball).

When the object is an elastic ball, the elastic coefficient of the object is smaller, and the parameter of B is smaller. The elastic ball selected in this experiment is shown in Figure 25. The force feedback wave of the haptic device along the x axis when Baxter is grasping the elastic ball is shown in Figure 26 and Figure 27 (robust control algorithm).

## 5. Results and Discussion

A new 6-DOF haptic device and an asymmetric teleoperation system are presented in this paper (as shown in Figure 5 and Figure 6), aiming at the tracking and transparency of the asymmetric teleoperation system; for single-joint PID control, the simulation experiment results showed that the performance of tracking is poor (as shown in Figure 10). A robust control algorithm based on the state feedback observer is proposed in Section 4 and simulation experiments was carried out on the new control algorithm. The results showed that the maximum values of displacement tracking errors in the three directions x, y, and z are 0.02 m, 0.01 m, and 0.015 m, respectively (as shown in Figure 14, Table 3). Compared with single-joint PID control, the performance of the new control algorithm is improved. Simulation experiments of force feedback were carried out, and Figure 13 shows the force feedback wave along the x axis of the master robot when grasping a solid object (no time delay). The force feedback wave fs is related to fm; Figure 14 shows the force feedback wave along the x axis of the master robot when grasping a solid object (constant time delay). The force feedback wave fs follows fm after a constant time delay; the equipment of the asymmetric teleoperation system are installed in the same room. Theoretically, there is no time-delay, and the time-delay can be ignored. Figure 15 shows the force feedback wave along the x axis of the master robot when grasping an elastic object (no time-delay). Figure 16 shows the force feedback wave when grasping an elastic object (constant time delay). We may see in Figure 15 and Figure 16 that the wave amplitude of fs is smaller than that of fm, for the object to be caught is an elastic ball, the elastic coefficient of the object is larger, and experiments on real asymmetric teleoperation system were carried out based on new control algorithm. The experimental results meet the tracking performance of the master–slave teleoperation system. Figure 21 shows the force feedback wave along the x axis of the master robot when grasping a solid object (no time delay), and Figure 22 shows the force feedback wave along the x axis of the master robot when grasping a solid object (constant time delay). It shows that the transparency of the asymmetric teleoperation system is consistent with the actual situation; when the object is an elastic ball, the elastic coefficient of the object is larger, the wave amplitude of fs is smaller than that of fm. When the object to be caught is an elastic ball, the results are consistent with the actual situation.

## 6. Conclusions

In this paper, a new 6-DOF haptic device and an asymmetric teleoperation system are presented. Aiming at the problems of tracking and transparency of the asymmetric teleoperation system, a new control algorithm was proposed; by analyzing simulation results between PID and a new control algorithm in the asymmetric teleoperation system, it is demonstrated that the performance of tracking is improved. Real tests were conducted to verify the correctness of the simulation results, and it could be seen from the test results that the new control algorithm is superior to PID. These indicators indicated that the new 6-DOF haptic device and an asymmetric teleoperation system meet the requirements. Moreover, the new 6-DOF haptic device could be extended to other teleoperation systems. The application of the teleoperation system is broad; in the future, more mathematical theories such as mechanism, kinematics, and dynamics will be applied in the teleoperation system, and the teleoperation system can be further applied in various fields. It has great application prospects and value.

## Figures and Tables

**Table 1:** Parameters of each rod of Baxter.

	m	r	l
1	5.7004	$\left[\begin{array}{ccc} 0.01783 & 0.00086 & 0.19127 \end{array}\right]$	$\left[\begin{array}{ccc} 0.04709 & 0.00013 & 0.00615\\ 0.00013 & 0.03767 & 0.00078\\ 0.00615 & 0.00078 & 0.003596 \end{array}\right]$
2	3.2269	$\left[\begin{array}{ccc} 0.06845 & 0.00268 & -0.00529 \end{array}\right]$	$\left[\begin{array}{ccc} 0.01175 & -0.00030 & 0.00207\\ -0.00030 & 0.02789 & -0.00019\\ 0.00207 & -0.00019 & 0.02079 \end{array}\right]$
3	4.31273	$\left[\begin{array}{ccc} -0.00276 & 0.00132 & 0.28286 \end{array}\right]$	$\left[\begin{array}{ccc} 0.02662 & 0.00029 & 0.0039\\ 0.00029 & 0.02844 & 0.00108\\ 0.00392 & 0.00108 & 0.01248 \end{array}\right]$
4	2.07206	$\left[\begin{array}{ccc} 0.00159 & -0.002611 & -0.01117 \end{array}\right]$	$\left[\begin{array}{ccc} 0.1318 & -0.00036 & -0.00019\\ -0.00036 & 0.00711 & 0.00108\\ -0.00019 & 0.00615 & 0.00926 \end{array}\right]$
5	2.24665	$\left[\begin{array}{ccc} -0.00168 & 0.0046 & 0.243111 \end{array}\right]$	$\left[\begin{array}{ccc} 0.01667 & 0.00018 & 0.00018\\ 0.00018 & 0.01675 & -0.00064\\ 0.00018 & -0.00064 & 0.00375 \end{array}\right]$
6	1.60979	$\left[\begin{array}{ccc} 0.00697 & -0.06041 & 0.006 \end{array}\right]$	$\left[\begin{array}{ccc} 0.00700 & -0.0044 & 0.00015\\ -0.00044 & 0.00387 & -0.00021\\ 0.00015 & -0.00021 & 0.00552 \end{array}\right]$
7	0.95093	$\left[\begin{array}{ccc} 0.00198 & 0.00125 & 0.134525 \end{array}\right]$	$\left[\begin{array}{ccc} 0.00025 & 0 & 0\\ 0 & 0.00027 & 0\\ 0 & 0 & 0.00031 \end{array}\right]$

**Table 2:** Setting Parameters.

	P	I	D
s0	28	16	24
s1	30	35	30
e0	62	5	0
e1	78	25	0
w0	5	2	1
w1	4	2	1
w2	3	0.4	0.2

**Table 3:** Trajectory tracking error in *x*, *y*, and *z* direction. (Robust control and PID control).

Direction	Robust Control	PID
*x*	0.02 m	0.1 m
*y*	0.01 m	0.045 m
*z*	0.015 m	0.028 m

## Data Availability

Not applicable.
